# Editorial: Foregrounding a rights-based agenda for sport events

**DOI:** 10.3389/fspor.2023.1282492

**Published:** 2023-10-02

**Authors:** David McGillivray, Gayle McPherson, Jason Bocarro, Daniela Heerdt

**Affiliations:** ^1^ Centre for Culture, Sport & Events, School of Business and Creative Industries, University of the West of Scotland, Paisley, United Kingdom; ^2^ College of Natural Resources, North Carolina State University, Raleigh, NC, United States; ^3^ Asser Instituut, Hague, Netherlands

**Keywords:** human rights, sport events, Olympics, FIFA World Cup, Commonwealth Games

**Editorial on the Research Topic** Foregrounding a rights-based agenda for sport events

Over the last decade, we have witnessed an explosion of academic literature and policy papers on major and mega sport events and human rights, fueled by media reporting of human rights abuses at various sporting events (e.g., Qatar 2022 FIFA World Cup). Even more recently, a number of systematic and scoping reviews have been undertaken, exploring the available literature on the topic area and outlining research priorities for those working in this space. We built on these trends to host a symposium, “Foregrounding a rights-based agenda for sport events” at the University of the West of Scotland in 2022, forming the genesis for this Research Topic. We wanted to publish papers that provided an overview of the state of the art on sport events and human rights while exploring specific rights-related issues associated with specific events. To that end, the Research Topic comprises five original research articles and a mini-review.

The Research Topic is introduced by a thoughtful mini-review from Heerdt (Addressing human rights abuses at mega-sporting events—a shared responsibility in theory and practice), following her keynote address at our 2022 symposium. In this review, Heerdt makes a case for a shared responsibility approach and argues that the adverse impacts of mega sport events on human rights can be minimized if such an approach is applied in a preventative and retrospective way and combined with the concept of collaborative remedy. Heerdt also stresses the importance of making positive changes sustainable, emphasizing that more academic research and better linkages with educational programs (at secondary and tertiary levels) will make this more achievable.

The remainder of this Research Topic focuses on specific rights contexts, with case studies of events and sport settings where human rights issues have been situated in the planning, delivery, or legacy of major and mega sport events. First, in the context of disability rights, Quinn and Misener (“It's classified: classification, disability rights and Commonwealth Games”) focus on the impact of the sport classification system on the integration of para-sport and athletes at the Commonwealth Games, with a specific focus on how lower resourced nations can be best supported to benefit from the integrated model that event follows. Second, Hanlon and Taylor (Workplace experiences of women with disability in sport organization) draw on an intersectional lens to highlight how women with a disability often experience barriers to employment and career progression in the hyper-masculinized sport sector. They analyze the lived experiences of eight women with varying types of disabilities working and volunteering in sport organizations in Victoria (Australia) and show that the individual's interpretation of being and feeling valued as a woman with a disability means not being judged against norms of ableism and trusting employers to provide supportive workplace adjustments as a matter of course rather than by exception.

Two further articles focus attention on other rights agendas around mega sport events. First, Oliver and De Lisio [Rights, not rescue: trafficking (in)securities at the sport mega-event] examine the impact of fantasies used to redevelop mega sport event cities on host communities, particularly related to the male-dominated FIFA World Cup and forced prostitution. They challenge existing narratives (event fantasies) in relation to humanitarian aid and the alleged involvement of women and children in forced labor and sexual exploitation in several FIFA host cities since the 2006 FIFA World Cup in Germany. They argue that the figure of the proverbial sex slave, as a highly racialized and hypersexualized trope, is mobilized through the sport mega-event to police the bodies of all women in labor and migration and put them at risk from the male gaze. Second, Bhimani and De Lisio (Sport mega-event fantasies to financialization: the case of Porto Maravilha) examine the relationship between sport mega-event construction and the financialization of housing in Rio de Janeiro, focusing on the area of Porto Maravilha, constructed prior to the 2016 Olympic Games. They draw on the work of Brazilian architect and author Raquel Rolnik to better understand the role of sport mega-event fantasies in the construction of Porto Maravilha—which they come to understand as a “speculative logic lubricant for finance.”

Finally, Islam’s paper (How has the Olympic legacy transformed the heart of East London? Understanding socio-economic exclusions and disproportionate COVID-19 impact on minoritised communities through a rights-based perspective) explores the experience of British–Bangladeshi and Black African Caribbean communities living in the areas surrounding London's Olympic Park. Islam argues that games-led regeneration has contributed to an unjust trade-off between pre-existing minoritized ethnic residents and wealthier gentrifiers, ignoring the real needs of the socially and economically disadvantaged ethnic minority communities in East London.

Theoretically, this collection of papers draws on various concepts to help understand and explain the phenomenon under study. Hanlon and Taylor draw on the Critical Disability Theory and Intersectionality to explore the workplace experiences of women with disabilities in sport organizations. In the same topic area (disability), Quinn and Misener explore various ableist theories to analyze the integration of para-athletes at the Commonwealth Games. Using this lens, they conclude that “classification within the context of Commonwealth Games has evolved to favor the most able, pushing out or rather ‘classifying out,’ athletes with higher support needs and greater impairment.” Islam builds on Lefebvre's “right to the city” and Purcell's right to participation when exploring socio-economic exclusions associated with the London 2012 Olympics. Finally, the articles of Oliver and De Lisio and Bhimani and De Lisio draw on theoretical frames of urban studies, including financialization and capital accumulation, when analyzing the FIFA World Cup and Olympic Games, respectively.

Overall, the contributions to this Research Topic illuminate some of the most important issues in the sport event and human rights landscape, many of which require further critical examination from both academic and non-academic actors. The papers in this collection also provide practical advice for sport event hosts and other key actors to prioritize rights in their planning and delivery. In summary, the Research Topic highlights that sport events can both help and hinder progress by foregrounding human rights and advocating for change. We must continue the debate to ensure we harness the potential of sport events to promote human rights. We hope that this Research Topic is shared widely with both academic and non-academic audiences so that those charged with the responsibility to enshrine rights-protecting practices in the bidding, planning, and delivery of sport events are drawing on complex, but nuanced, guidance to ensure that these mega projects are rights-enhancing and not rights-infringing.

